# Synthesis, Characterization And Antitumor Activity Of Copper(II) Complexes, [CuL_2_] [HL^1-3^=N,N-Diethyl-N'-(R-Benzoyl)Thiourea (R=H, o-Cl and p-NO_2_)]

**DOI:** 10.1155/BCA.2005.299

**Published:** 2005

**Authors:** Wilfredo Hernández, Evgenia Spodine, Lothar Beyer, Uwe Schröder, Rainer Richter, Jorge Ferreira, Mario Pavani

**Affiliations:** 1 Universidad de Chile CIMAT Facultad de Ciencias Químicas y Farmacéuticas Casilla 233 Santiago 1 Chile; 2 Universität Leipzig, Institut für Anorganische Chemie Johannisallee 29 Leipzig D-04103 Germany; 3 Universidad de Chile Instituto de Ciencias Biomédicas Programa de Farmacología Molecular y Clínica Facultad de Medicina, Casilla 233 Santiago 1 Chile

**Keywords:** Copper(II) complexes, Acylthiourea, Antitumor, cytotoxic activity, Cell growth, Cellular DNA

## Abstract

The copper (II) complexes (CuL_2_) were prepared by reaction of Cu(CH_3_COO)_2_ with the corresponding derivatives of acylthioureas in a Cu:HL molar ratio of 1:2. Acylthiourea ligands, N,N-diethyl-N'-(R-benzoyl) thiourea (HL^1-3^) [R=H, o-Cl and p-NO_2_] were synthesized in high yield (78-83%) and characterized by elemental analysis, infrared spectroscopy, ^1^H and ^13^C NMR spectroscopy. The complexes CuL_2_ were characterized by elemental analysis, IR, FAB(+)-MS, magnetic susceptibility measurements, EPR and cyclic voltammetry. The crystal structure of the complex Cu(L^2^)_2_ shows a nearly square-planar geometry with two deprotonated ligands (L) coordinated to Cu^II^ through the oxygen and sulfur atoms in a *cis* arrangement. The antitumor activity of the copper(II) complexes with acylthiourea ligands was evaluated *in vitro* against the mouse mammary adenocarcinoma TA3 cell line. These complexes exhibited much higher cytotoxic activity (IC_50_ values in the range of 3.9-6.9 μM) than their corresponding ligands (40-240 μM), which indicates that the coordination of the chelate ligands around the Cu^II^ enhances the antitumor activity and, furthermore, this result confirmed that the participation of the nitro and chloro substituent groups in the complex activities is slightly relevant. The high accumulation of the complexes Cu(L^2^)_2_ and Cu(L^3^)_2_ in TA3 tumor cells and the much faster binding to cellular DNA than Cu(L^1^)_2_ are consistent with the *in vitro* cytotoxic activities found for these copper complexes.


### Synthesis, Characterization And Antitumor Activity Of Copper(II) Complexes, $[CuL_2]$ [$HL^{1-3}$=N,N-Diethyl-N′-(R-Benzoyl)Thiourea (R=H, o-Cl and p-$NO_2$)]

Wilfredo Hernández [a], Evgenia Spodine [a,*], Lothar Beyer [b], Uwe Schröder [b], Rainer Richter [b], Jorge Ferreira [c], Mario Pavani [c]

[a] *Universidad de Chile, CIMAT, Facultad de Ciencias Químicas y Farmacéuticas, Casilla 233, Santiago 1, Chile*

[b] *Universität Leipzig, Institut für Anorganische Chemie, Johannisallee 29, D-04103 Leipzig, Germany.*

[c] *Universidad de Chile, Instituto de Ciencias Biomédicas, Programa de Farmacología Molecular y Clínica, Facultad de Medicina, Casilla 233, Santiago 1, Chile.*

**GRAPHICAL ABSTRACT**

The copper(II) complexes of acylthiourea derivatives have been synthesized and characterized by elemental analysis, FT-IR, FAB(+)-MS, NMR($^1H$, $^{13}C$), magnetic measurements and cyclic voltammetry. Assessment of antitumor activity against the mouse mammary adenocarcinoma TA3 cell line has demonstrated that all $Cu^{II}$ complexes were more active than their respective ligands and $Cu(L^3)_2$ showed a higher cytotoxicity than all the compounds tested. Cellular copper accumulation and DNA binding were also determined and the results are consistent with the cytotoxic activities obtained.

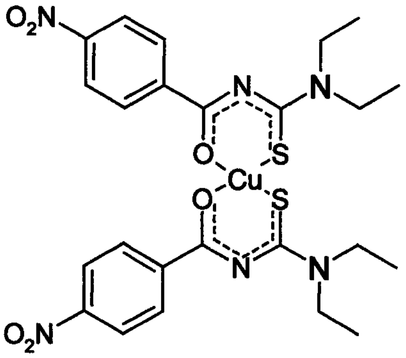

$Cu(L^3)_2$

* Corresponding author. Tel.:+56-2-9782862; fax:+56 -2-978-2868; e-mail: espodine@ciq.uchile.cl

### ABSTRACT

The copper (II) complexes ($CuL_2$) were prepared by reaction of $Cu(CH_3COO)_2$ with the corresponding derivatives of acylthioureas in a Cu:HL molar ratio of 1:2. Acylthiourea ligands, N,N-diethyl-N′-(R-benzoyl)thiourea ($HL^{1-3}$) [R=H, o-Cl and p-$NO_2$] were synthesized in high yield (78-83%) and characterized by elemental analysis, infrared spectroscopy, $^1H$ and $^{13}C$ NMR spectroscopy. The complexes $CuL_2$ were characterized by elemental analysis, IR, FAB(+)-MS, magnetic susceptibility measurements, EPR and cyclic voltammetry. The crystal structure of the complex $Cu(L^2)_2$ shows a nearly square-planar geometry with two deprotonated ligands (L) coordinated to $Cu^{II}$ through the oxygen and sulfur atoms in a *cis* arrangement. The antitumor activity of the copper(II) complexes with acylthiourea ligands was evaluated *in vitro* against the mouse mammary adenocarcinoma TA3 cell line. These complexes exhibited much higher cytotoxic activity ($IC_{50}$ values in the range of 3.9-6.9 μM) than their corresponding ligands (40-240 μM), which indicates that the coordination of the chelate ligands around the $Cu^{II}$ enhances the antitumor activity and, furthermore, this result confirmed that the participation of the nitro and chloro substituent groups in the complex activities is slightly relevant. The high accumulation of the complexes $Cu(L^2)_2$ and $Cu(L^3)_2$ in TA3 tumor cells and the much faster binding to cellular DNA than $Cu(L^1)_2$ are consistent with the *in vitro* cytotoxic activities found for these copper complexes.

**Keywords:** Copper(II) complexes; Acylthiourea; Antitumor / cytotoxic activity; Cell growth; Cellular DNA.

### 1. INTRODUCTION

Whilst most of the investigations in the treatment of cancer diseases are oriented to synthesize new metal complexes analogous to *cis*-diamminedichloroplatinum(II) (cisplatin) (antineoplastic agent of clinical use) /1, 2/, there is a growing number of non-platinum metal complexes which also exhibit remarkable anticancer activities /3-5/. Thus, the bis(acetate) bis(imidazole)copper(II) complex has shown a high cytotoxic activity against to the mouse B16 melanoma cancer cell line, and determinations realized with ϕ X174 RF DNA indicate that the target of this complex may be the guanine residues of the DNA helix /6/. In addition, the copper (II) complexes with thiosemicarbazone derivatives (O,N,S-tridentate chelate ligands) are well-known by their biological applications as antiviral /7/, antimicrobial /8/, antitubercular and antitumor agents /9-11/. In this sense, Antholine *et al.* /12, 13/ have reported that the copper complex [{$CuL(MeCO_2)$}$_2$] (HL=2-formylpyridine thiosemicarbazone) and related compounds have marked antitumor activities, being more potent than the free ligands against Ehrlich cells injected into mice /14/, Sarcoma 180 ascites tumors /15/ and Chinese hamster ovary cells /16, 17/. On the other hand, the biological activities of complexes with thiourea derivatives have been successfully screened for various biological actions /18-21/. Recently, Xu Shen *et al.* /22/ have reported the antitumor activities of the $Mn^{II}$ complex derived from the desulfurization and hydrolysis of the ligand, N-(p-nitrophenyl)-N′-(methoxycarbonyl) thiourea. This complex showed high cytotoxicity *in vitro* at micromolar concentrations against human ovary tumor 3 AO and mice P388 *in vitro.* In our previous work, we reported the *in vitro* antitumor activity of the platinum (II) complexes with analogous ligands against mouse mammary adenocarcinoma TA3 cells, where these complexes showed to be more cytotoxic at low micromolar concentrations than their corresponding ligands /23/. Since our earlier work had revealed that platinum complexation with acylthiourea ligands enhances the antitumor activity, we were motivated to use copper as different central atom in order to obtain a cytotoxic behaviour similar to the platinum complexes mentioned before. In the present work, we report about synthesis and characterization of copper(II) complexes with ligands N,N-diethyl-N′-R-benzoylthiourea [R=H, o-Cl and p-$NO_2$] and their *in vitro* cytotoxic effect, copper accumulation and binding to cellular DNA on mouse TA3 mammary adenocarcinoma.

### 2. EXPERIMENTAL

### 2.1. Materials

Copper(II)acetate monohydrate, sodium sulphate, sodium bicarbonate, benzoylchloride, o-chlorobenzoylchloride, p-nitrobenzoylchloride and diethylamine from Aldrich were used as received. All solvents were reagent grade and were purified by standard procedures before use.

Eagle's Minimum Essential Medium [MEM(E)] was supplied by Sigma (USA) and the fetal bovine serum [FBS] was provided by Difco (Detroit, MI). The penicillin, streptomycin and sodium chloride 0.9% were obtained from Sanderson's laboratory (Chile).

### 2.2. Measurements

Elemental analyses were carried out on a Fisons-Carlo Erba 1108 elemental microanalyser. Melting points were determined on a Boetius melting-point apparatus. Magnetic susceptibilities at room temperature (296 K) were measured using the Gouy method on a Johnson-Mathey MSB-MKI balance with $HgCo(NCS)_4$ as standard and diamagnetic corrections were made using Pascal 's constants /24/. The infrared (IR) spectra were recorded in solid state (KBr pellets) on a Bruker FT-IR IFS 55 Equinox spectrophotometer in the range 4000 - 400 $cm^{-1}$. FAB(+) mass spectra were obtained on a ZAB-HSQ (V.G. Analytical Ltd.) spectrometer. Determinations of copper concentration in biological samples were performed on a Perkin Elmer 3110 atomic absorption spectrometer. NMR ($^1H$ and $^{13}C$) spectra of the ligands were recorded on a Bruker Advance DRX-300, using $CDCl_3$ as solvent and TMS as internal standard. Cyclic voltammetric measurements were made in DMSO solvent on a Bioanalytical System BAS CV-50 W with an X-Y recorder using a carbon disk (6 mm diameter) as working electrode vs. SCE as reference and a Pt filament as auxiliary electrode. The solutions of the complexes ($1 \times 10^{-3}$ M ) in DMSO with tetrabutylammonium perchlorate (TBAP) (0.1 M) as supporting electrolyte were deoxygenated by a stream of dry nitrogen for at least 10 min. EPR spectra of the copper complexes solutions in $CH_2Cl_2$ at room temperature were measured using a Bruker ESP 300 E spectrometer operating at X-band frequency.

### 2.3. Synthesis of the ligands

The ligands N,N-diethyl-N′-(R-benzoyl)thiourea (HL[1], HL[2] and HL[3]) were prepared according to the reported methods /25, 26/ as shown in Scheme 1.

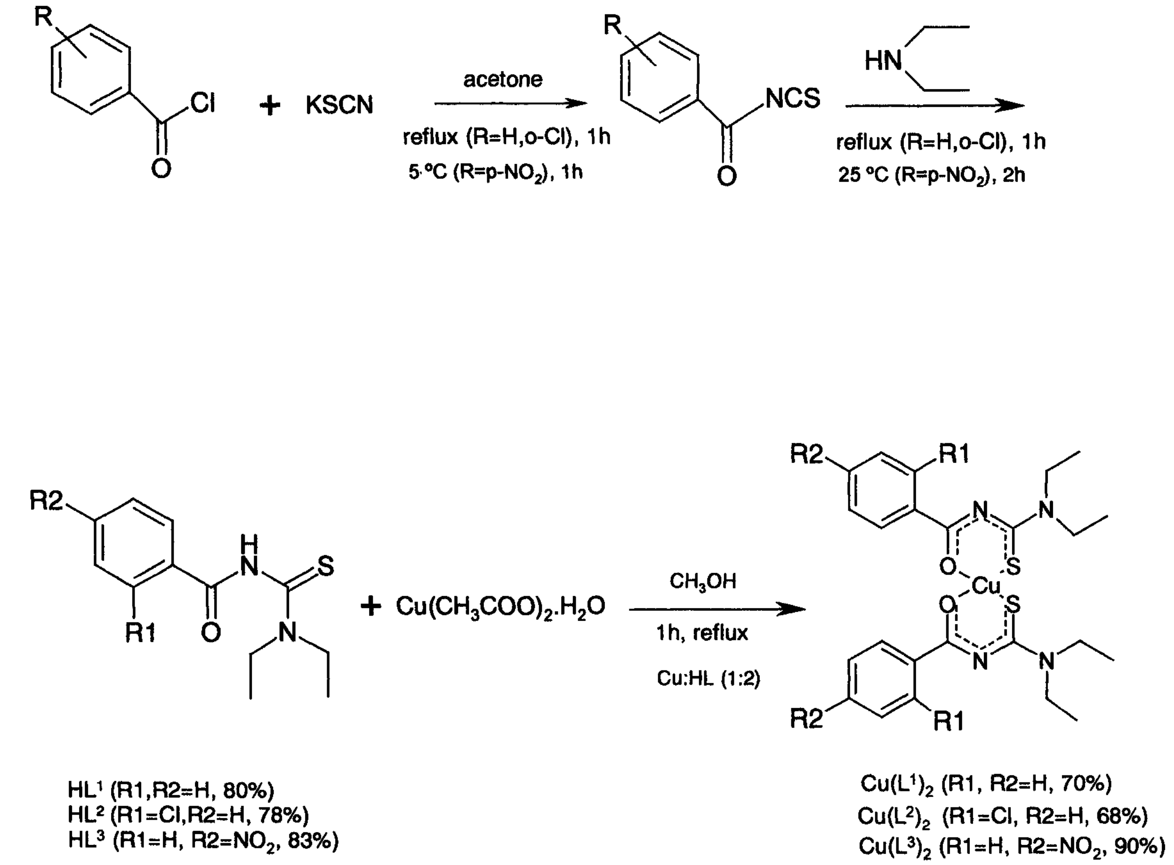

**Scheme 1.** Synthesis of the ligands N,N-diethyl-N′-R-benzoylthiourea and their copper(II) complexes.

### *2.3.1. N,N-Diethyl-N′-benzoylthiourea (HL[1])*

Needle-shaped pale yellow crystals. Yield: 80.0 %, m.p. 98-100°C. Anal. Calc. for $C_{12}H_{16}ON_2S$ (236.0 g/mol): C, 61.0%; H, 6.8%; N, 11.85%; S, 13.6%. Found: C, 61.6%; H, 6.8%; N, 11.6%; S, 13.4%. IR (KBr, $cm^{-1}$): ν(N-H) 3200 $cm^{-1}$ (br,sh), ν(C=O) 1690 $cm^{-1}$ (vs), ν(C=S) 1232 (s). $^1$H-NMR (300 MHz, $CDCl_3$): δ 1.30 (t, 3H, $CH_3$), 1.37 (t, 3H, $CH_3$), 3.63 (q, 2H, $CH_2$), 4.04 (q, 2H, $CH_2$), 7.48 (t, $2H_{meta}$, Ph), 7.58 (t, $1H_{para}$, Ph), 7.84 (d, $2H_{ortho}$, Ph), 8.35 (s, 1H, NH). $^{13}$C-NMR (75.5 MHz, $CDCl_3$): δ 10.5, 12.3 ($CH_3$); 46.7, 47.0 ($CH_2$); 126.8, 127.9, 131.9, 133.6 (ortho, meta, para, i-Ph); 162.8 (C=O), 178.2 (C=S).

### *2.3.2. N,N-Diethyl-N′-(o-chlorobenzoyl)thiourea (HL[2])*

Needle-shaped white crystals. Yield: 78.0 %, m.p. 115-117 °C. Anal. Calc. for $C_{12}H_{15}ON_2SCl$ (270.8 g/mol): C, 47.78%; H, 4.68%; N, 9.29%; S, 10.63%; Cl, 11.76%. Found: C, 48.0%; H, 4.89%; N, 9.42%; S,

10.7%; Cl, 12.04%. IR (KBr, $cm^{-1}$): ν(N-H) 3240 $cm^{-1}$ (br, m), ν(C=O) 1655 $cm^{-1}$ (vs), ν(C=S) 1233 (s). $^1$H-NMR (300 MHz, $CDCl_3$): δ 1.34 (t, 6H, $CH_3$), 3.69 (q, 2H, $CH_2$), 3.97 (q, 2H, $CH_2$), 7.24 (d, 1$H_{meta}$, o-Cl-Ph), 7.29 (t, 1$H_{meta}$, o-Cl-Ph), 7.35 (t, 1$H_{para}$, o-Cl-Ph), 7.74 (d, 1$H_{ortho}$, o-Cl-Ph), 8.32 (br, 1H, NH). $^{13}$C-NMR (75.5 MHz, $CDCl_3$): δ 12.5, 13.3 ($CH_3$); 45.9, 46.9 ($CH_2$); 129.5, 130.7, 132.6, 134.3 (ortho, meta, para, i-Ph); 167.3 (C=O), 169.5 (C=S).

### 2.3.3. *N,N-Diethyl-N′-(p-nitrobenzoyl)thiourea ($HL^3$)*

Square yellow crystals. Yield: 83.0 %, m.p. 163-165 °C. Anal. Calc. for $C_{12}H_{15}O_3N_3S$ (281 g/mol): C, 51.25%; H, 5.34%; N, 14.95%; S, 11.39%. Found: C, 50.91%; H, 5.38%; N, 14.88%; S, 10.96%. IR (KBr, $cm^{-1}$): ν(N-H) 3284 $cm^{-1}$ (br, m), ν(C=O) 1648 $cm^{-1}$ (vs), ν(C=S) 1230 (s). $^1$H-NMR (300 MHz, $CDCl_3$): δ 1.31 (t, 3H, $CH_3$), 1.36 (t, 3H, $CH_3$), 3.61 (q, 2H, $CH_2$), 4.03 (q, 2H, $CH_2$), 8.01 (d, 2$H_{ortho}$, p-$NO_2$Ph), 8.34 (d, 2$H_{meta}$, p-$NO_2$-Ph), 8.41 (s, 1H, NH). $^{13}$C-NMR (75.5 MHz, $CDCl_3$): δ 11.8, 13.7 ($CH_3$); 48.2 ($CH_2$); 124.4, 129.4, 138.5, 140.9 (ortho, meta, para, i-Ph); 162.4 (C=O), 178.8 (C=S).

### 2.4 Synthesis of the copper(II) complexes

The complexes were prepared according to the following general procedure.

To a stirred solution of N,N-diethyl-N′-(R-benzoyl)thiourea (1 mmol) in methanol (30 mL) was added dropwise a solution of $Cu(CH_3COO)_2 \cdot H_2O$ (0.5 mmol) in methanol (30 mL). The reaction mixture was refluxed for 1 h and then stirred at room temperature for 24 h. The green solution was filtrated and the filtrate was evaporated to dryness under reduced pressure to yield a dark green solid . The solid was washed several times with small portions of cold ethanol and dried in vacuo.

### 2.4.1. *Bis(N,N-diethyl-N′-benzoylthioureato)copper(II), $Cu(L^1)_2$*

Green solid. Yield: 70.0 %, m.p. 118-120 °C. Anal. Calc. for $C_{24}H_{30}O_2N_4S_2Cu$ (533.5 g/mol): C, 54.0%; H, 5.6%; N, 10.5%; S, 12.0%. Found: C, 53.9%; H, 5.4%; N, 10.7%; S, 11.9%. IR (KBr, $cm^{-1}$): ν(C=O) 1580 $cm^{-1}$ (w), ν(C=S) 1212 (m). FAB(+)-MS (matrix: 3-NBA): m/z 534.3 ($[M+H]^+$, 100%), 297.3 ($[M-L]^+$, 57%).

### 2.4.2. *Bis(N,N-diethyl-N′-(o-chlorobenzoyl)thioureato)copper(II), $Cu(L^2)_2$*

Dark green prisms. Yield: 0.205 g (68 %), m.p. 126-128 °C. Anal. Calc. for $C_{24}H_{28}O_2N_4S_2Cl_2Cu$ (603.08 g/mol): C, 47.79%; H, 4.68%; N, 9.29%; S, 10.63%; Cl, 11.76%. Found: C, 48.0%; H, 4.89%; N, 9.42%; S, 10.7%; Cl, 11.98%. IR (KBr, $cm^{-1}$): ν(C=O) 1589 $cm^{-1}$ (w), ν(C=S) 1206 (m). FAB(+)-MS (matrix: 3-NBA): m/z 604 ($[M+H]^+$, 100%), 332.5 ($[M-L]^+$, 47.4%),

### 2.4.3. *Bis(N,N-diethyl-N′-(p-nitrobenzoyl)thioureato)copper(II), $Cu(L^3)_2$*

Green solid. Yield: 0.28 g (90 %), m.p. 208-210 °C . Anal. Calc. for $C_{24}H_{28}O_6N_6S_2Cu$ (624.18 g/mol): C, 46.19%; H, 4.49%; N, 13.47%; S, 10.29%. Found: C, 45.61%; H, 4.63%; N, 13.39%; S, 10.27%. IR (KBr, $cm^{-1}$): ν(C=O) 1625 $cm^{-1}$ (w), ν(C=S) 1205 (m). FAB(+)-MS (matrix: 3-NBA): m/z 624 ($M^+$, 86.14%); 613.1 (3-NBA, 100%), 342.5 ($[M-L]^+$, 25.6%).

### 2.5. Crystal structure determination

The crystallographic data of **$Cu(L^2)_2$** were obtained on a Siemens CCD Smart Diffractometer (Mo Kα radiation, λ=0.71073 Å, graphite monochromator, T= 218(2) K). The intensities were corrected for Lorentz and polarization effects and for absorption using SADABS. The structure was solved by direct methods, which revealed the positions of all non-hydrogen atoms and refined on $F^2$ by a full-matrix least-squares procedure using anisotropic displacement parameters. The hydrogen atoms were located from difference Fourier syntheses and refined isotropically. All calculations were carried out using the SHELXS-97 and SHELXL-97 programs /27-28/. Crystal data collection and refinement details for the complex **$Cu(L^2)_2$** are summarized in Table 1.

**Table 1**

Crystal data and refinement summary for the copper(II) complex $Cu(L^2)_2$.

| | |
|---|---|
| Empirical formula | $C_{24}H_{28}O_2N_4S_2Cl_2Cu$ |
| Formula weight ($g{\cdot}mol^{-1}$) | 603.06 |
| Crystal habit, color | dark green prisms |
| Crystal system | triclinic |
| Space group | P-1 |
| a (Å) | 10.896(1) |
| b (Å) | 11.194(1) |
| c (Å) | 10.401(2) |
| α (°) | 88.154(3) |
| β (°) | 85.751(4) |
| γ (°) | 81.193(3) |
| Volume (Å$^3$) | 1370.0(3) |
| Z | 2 |
| Density (calc.) ($g{\cdot}cm^{-3}$) | 1.462 |
| Crystal size (mm) | 0.29 x 0.32 x 0.48 |
| Absorption coeff., $\mu$(Mo-$K_\alpha$)/( $mm^{-1}$) | 1.173 |
| F(000) | 622 |
| 2θ Range (°) | 3.8 – 57.9 |
| Measured reflections | 6482 |
| Unique reflections | 5651 |
| Observed reflections – (I> 2σ (I)) | 4781 |
| Refined parameters | 428 |
| $wR_2$ (unique refl.) | 0.0805 |
| $R_1$ (observed refl.) | 0.0290 |
| Goodness-of-fit on $F^2$ | 1.034 |
| Largest difference peak and hole (e Å$^{-3}$) | 0.34/-0.28 |

### 2.6. Biological evaluation

### 2.6.1. *Culture of cells and cytotoxic assay*

The mouse mammary adenocarcinoma TA3 cell line was obtained from ascites fluid of young adult male CAF 1 Jax mice. The cells were cultured at 37 °C in a growth medium consisting of Eagle's Minimum Essential Medium [MEM(E)] with Earle's Salts, L-glutamine and 25 mM HEPES, supplemented with 10% fetal bovine serum [FBS], 44 mM sodium bicarbonate, 100 U $mL^{-1}$ penicillin and 100 μg $mL^{-1}$ streptomycin.

For these experiments, cells at a concentration of 2 x $10^5$ $mL^{-1}$ were seeded by 96 h in 20 mL of culture medium /29/. After 24 h of tumor cell growth, the compounds were dissolved in DMSO just before the experiments and a calculated amount of drug solution was added to each growth medium to obtain the desired concentrations of either the ligands ( 20, 40 and 60 μM) or their copper complexes (1, 5 y 10 μM). The final DMSO concentration in the culture medium, 0.5%, did not show an appreciable effect on cell growth for these assays. Parallel cultures were used as control. Viable cells were determined using a Neubauer counting chamber every 24 h. The concentrations of every compound were plotted with respect to the percentage of cell survival. The $IC_{50}$ values were obtained by graphical interpolation at 48 h of exposure on each one of the compounds. These values represent the drug concentration (μM) required to inhibit cell growth by 50%. All assays were performed in triplicate cultures and repeated three times in independent pattern.

### 2.6.2. *Cellular copper accumulation*

Cells (5 x $10^6$ / mL) were incubated at 37 °C with each one of the copper complexes (125 μM). After various time intervals, an aliquot of the mixture was removed and assayed according to the reported method /30/. Briefly, the cells were pelleted by centrifugation and washed three times with ice-cold PBS (0.1 M phosphate buffer in 0.15 M NaCl, pH 7.4). Pellets were dried and digested overnight in concentrated nitric acid, then diluted with distilled water to give a final $HNO_3$ concentration of 20%. Cellular copper concentrations were measured by atomic absorption spectroscopy and the amount of accumulated Cu was expressed as nmol Cu/$10^6$ cells.

### 2.6.3. *Binding to cellular DNA in TA3*

Each one of the copper complexes (20 –100 μM) was incubated with the TA3 tumor cells (5 x $10^6$ /mL ) at 37 °C for 2 h of exposure. Then, cells were isolated by centrifugation, suspended in 1 mL of PBS and centrifuged again. This washing step was repeated twice. After centrifugation cells were taken up in 500 μL of TEN buffer, pH = 8 (10 mM Tris, 10 mM EDTA and 150 mM NaCl), and subsequently 5 μL of proteinase K and 50 μL of 10% SDS were added. Lysed cells were kept at 55 °C for 1 h. Proteins were then removed by chloroform/phenol (1:1) extraction followed by an extraction with chloroform alone. DNA was precipitated from the aqueous layer by adding an equal volume of isopropyl alcohol. Finally, precipitated DNA was removed from the solution, washed with 70% ethanol and dissolved in 1 mL of water /31/.

The DNA concentration was determined by measuring the UV absorption at 260 nm and concentration of base pairs was calculated using mean molar extinction coefficient per base pair $\varepsilon_{260}$ = 16800 $M^{-1}$ $cm^{-1}$. Copper concentration was measured by FAAS. From this information, the $r_b$ values (the number of drug molecules bound per base) were calculated.

### 3. RESULTS AND DISCUSSION

### 3.1. Synthesis

The synthesis of the copper(II) complexes and their ligands is shown in Scheme 1. The acylthiourea ligands, N,N-diethyl-N′-R-benzoylthiourea (**$HL^1$** , **$HL^2$** and **$HL^3$**) were prepared according to the methods described by Hartmann /25/ and Brindley /26/. The synthesis involves the reaction of R-benzoyl chloride with potassium thiocyanate in acetone followed by reacting R-benzoyl isothiocyanate with diethylamine. For the synthesis of **$HL^3$**, a modification of Brindley 's method was carried out; the solvent used was acetone instead of acetonitrile and the intermediary product, isothiocyanate, was not removed from the reaction mixture. With these changes it was possible to obtain a high yield, which was compared with those obtained for related acylthioureas described in the literature /32, 33/. The ligands **$HL^1$** and **$HL^2$** were recrystallized from mixed solvents of $CH_2Cl_2$-$CH_3OH$ (2:1, v/v) and $CHCl_3$-EtOH (2:1, v/v), respectively, whereas **$HL^3$** was recrystallized from $CH_2Cl_2$. The ligands were obtained in satisfactory yield (78-83 %) and characterized by elemental analysis, infrared spectroscopy and $^1H$ and $^{13}C$ NMR spectroscopy.

The copper (II) complexes **$Cu(L^1)_2$**, **$Cu(L^2)_2$** and **$Cu(L^3)_2$** were prepared by refluxing the methanolic mixture of the ligand (HL) and $Cu(CH_3COO)_2 \cdot H_2O$ in the molar ratio of 2:1. With this employed technique, the yields of copper(II) complexes were improved as related to other methods that carry out the synthesis during several days at room temperature /34/. Recrystallization of the complex **$Cu(L^2)_2$** from hot ethanol yielded crystals suitable for structural determination by X-ray diffraction. All three copper (II) complexes were characterized by elemental analysis and IR, FAB(+)-mass, magnetic susceptibility, EPR and cyclic voltammetry. The elemental and spectroscopic analysis of the ligands and their copper (II) complexes are consistent with the proposed structures given in Scheme 1. In general, the methods used for the preparation of these compounds were adopted because they are fast, easy and give higher yields than other methods, which contain some modifications in the synthetic route, such as, temperature, reaction time, solvents and separation procedures of the product.

### 3.2. IR and $^1$H-NMR spectra

In the IR spectra, all acylthiourea ligands exhibited NH stretching bands in the range of 3200 – 3284 $cm^{-1}$, which disappeared after coordination. This indicates the loss of the proton originally bonded to nitrogen atom of the (NH-CO) amide group. The vibrational frequencies due to the carbonyl (1648-1690 $cm^{-1}$) and thiocarbonyl (1230-1233 $cm^{-1}$ ) groups in the free ligands are shifted (65-68 and 21-25 $cm^{-1}$, respectively) towards lower frequencies upon complexation, confirming that the deprotonated ligands are coordinated to $Cu^{II}$ ion through the oxygen and sulfur donor atoms /35-37/.

In the $^1$H-NMR spectra for the ligands, the presence of the chloro- and nitro- substituent groups on the benzoyl moiety (**$HL^2$** and **$HL^3$**), causes no significant changes in the chemical shifts of the N-H group (8.32

and 8.41 ppm, respectively) relative to the ligand **HL**[1] (8.35 ppm) with unsubstituted benzoyl moiety. The aromatic protons signals (ortho and meta ) are shifted upfield (0.49 and 0.24 ppm, respectively) for **HL**[2] and downfield (0.17 and 0.86 ppm, respectively) for **HL**[3] with respect to **HL**[1]. These results are consistent with the mesomeric/inductive effects expected for these substituents. On the other hand, the resonances of the methylene protons bonded to the nitrogen of the respective thioamide group appeared as two separated signals at 3.61-3.69 and 3.97-4.04 ppm, respectively, showing in each signal a well-resolved quartet. The magnetic inequivalence of the $CH_2$ protons can be attributed to the restricted rotation around the C-N bond between the thiocarbonyl group and the amine nitrogen due to the partial double character of this bond /38, 39/.

### 3.3. Magnetic data and EPR spectra

The room temperature magnetic moments for the copper (II) complexes **Cu(L$^1$)$_2$**, **Cu(L$^2$)$_2$** and **Cu(L$^3$)$_2$** resulted to be 2.0, 1.93 and 1.82 BM, respectively. These values are consistent with the presence of one unpaired electron in mononuclear $d^9$ copper (II) complexes. Furthermore, these magnetic data are in agreement with those of the *cis*-bis(acylthioureato) copper (II) complexes ($\mu_{eff}$= 1.8-2.00 BM) reported in the literature /32, 40/.

The X-band EPR spectra for these copper(II) complexes were determined in $CH_2Cl_2$ solution at room temperature. All three spectra exhibit four well-resolved absorption lines ($I_{cu}$=3/2) with isotropic parameters ($g_{iso}$= 2.083-2.084 and $A_{iso}^{Cu}$ = 79.8-80.4 G) and these results are similar to those of previously reported copper(II) complexes of square-planar geometry which contain acylthiourea and imidoylthiourea ligands ($g_{iso}$= 2.081-2.082; $A_{iso}^{Cu}$ = 63.0-78.0 G; T=293K, $CHCl_3$) /41-44/.

### 3.4 Electrochemical studies

The redox processes of the copper (II) complexes were electrochemically confirmed by cyclic voltammetry. The voltammograms of all free ligands do not present any oxidation or reduction peak in the potential range studied, except for the ligand **HL**[3], which show a reduction peak at –1.05 V, which may correspond to the reduction of the p-nitrophenyl group. Cyclic voltammograms of the copper (II) complexes in DMSO show a quasi-reversible voltammetric response for the Cu(II)/Cu(I) couple with reduction peaks at –0.30, -0.23 and –0.21 V, and $\Delta E_p$ values (where $\Delta E_p$ =/$E_{pc}$-$E_{pa}$/) of 89, 95 and 91 mV for the complexes **Cu(L$^1$)$_2$**, **Cu(L$^2$)$_2$** and **Cu(L$^3$)$_2$** (Figure 2a) , respectively. The ratio of anodic ($i_{pa}$) and cathodic ($i_{pc}$) peak currents is in the range of 0.83-0.91 at 100 $mVs^{-1}$ scan rate. The $\Delta E_p$ values for these complexes were lower than those of related copper complexes ($\Delta Ep$ >200 mV in DMF) considered in the literature as irreversible processes /34/. Furthermore, as we can observe in Figure 2b, the cyclic voltammogram for the complex **Cu(L$^3$)$_2$** shows that the metal-based potential peaks change with the scan rate (50-500 mV $s^{-1}$) indicating that the redox process becomes more irreversible as reported for the related copper complexes too /40/.

The presence of the nitro and chloro substituent groups on the benzoyl moiety has a weak influence on the electrochemical properties of these copper (II) complexes /32/. Only small differences found in the cathodic potentials indicate that **Cu(L$^1$)$_2$** must be more difficult to reduce than **Cu(L$^2$)$_2$** and **Cu(L$^3$)$_2$** with the chloro and nitro substituent groups on the benzoyl moiety.

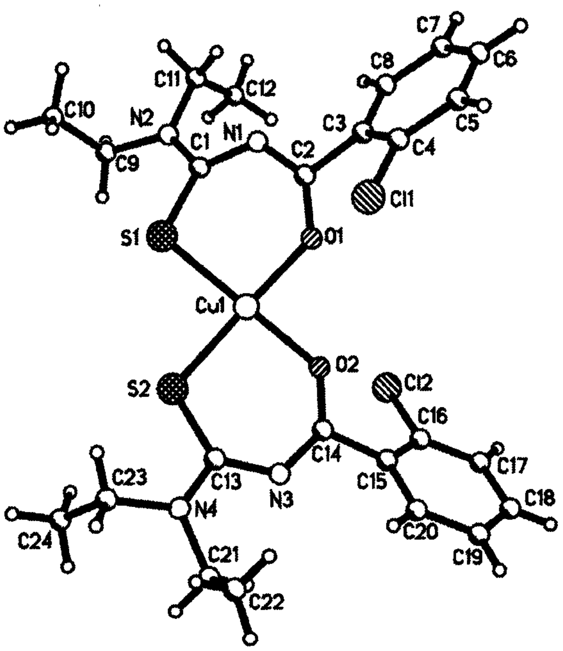

**Fig. 1:** Molecular structure of Cu(L$^2$)$_2$ (50 % thermal ellipsoids).

### 3.5. Structural data

The molecular structure of the complex **Cu(L$^2$)$_2$**, together with the atom numbering scheme adopted, is shown in Fig. 1. Selected bond distances and bond angles are listed in Table 2.

As can be seen from Fig. 1, the structure shows that the copper(II) ion has a slightly distorted square-planar geometry indicated by the angle between the planes Cu1S1O1 and Cu1S2O2 of 12.1(1)°. The structural determination of this complex confirms that two deprotonated ligands are coordinated bidentately to the copper(II) ion through the sulphur and oxygen donor atoms in a *cis* arrangement /44,45/. The coordination around the Cu$^{II}$ ion generates two six-membered chelate rings, which form angles with the adjacent phenyl groups of 48.2(1) and 43.0(1)°. This result probably indicates that the presence of the chloro substituent on the benzoyl moiety leads to a steric repulsion between chelate rings and phenyl rings.

By comparison with the reported structure of the free ligand **HL$^2$** /46/, the bond lengths of the thiocarbonyl (S1-C1 1.738(2), S2-C13 1.735(2) Å) and carbonyl (O1-C2 1.261(3), O2-C14 1.264(2) Å) moieties in the complex **Cu(L$^2$)$_2$** are longer than in its ligand (S=C 1.658(2), O=C 1.218(2) Å) whereas the N-C bond distances in this complex (N1-C1 1.353(3)/N3-C13 1.350(2) and N1-C2 1.320(3)/N3-C14 1.325(2) Å) are shorter compared to **HL$^2$** (N1-C1 1.428(2) and N1-C2 1.360(2) Å). This indicates that the decrease of the bond orders of the thiocarbonyl and carbonyl groups together with the changes in the N-C bond lengths on coordination, originates from a π-electron delocalization over the chelate ring of the complex **Cu(L$^2$)$_2$** /33,46/.

**Table 2**

Selected bond lengths (Å) and angles (°) for $Cu(L^2)_2$

| | | | |
|---|---|---|---|
| **Bond Lengths** | | | |
| Cu1-S1 | 2.2512(7) | Cu1-S2 | 2.2353(6) |
| Cu1-O1 | 1.934(1) | Cu1-O2 | 1.932(1) |
| S1-C1 | 1.738(2) | S2-C13 | 1.735(2) |
| O1-C2 | 1.261(3) | O2-C14 | 1.264(2) |
| N1-C1 | 1.353(3) | N3-C13 | 1.350(3) |
| N1-C2 | 1.320(3) | N3-C14 | 1.325(2) |
| N2-C1 | 1.326(3) | N4-C13 | 1.338(2) |
| Cl1-C4 | 1.746(2) | Cl2-C16 | 1.739(2) |
| **Bond Angles** | | | |
| S1-Cu1-O1 | 93.77(4) | S2-Cu1-O2 | 92.99(4) |
| Cu1-S1-C1 | 102.73(7) | Cu1-S2-C13 | 105.38(6) |
| Cu1-O1-C2 | 129.7(1) | Cu1-O2-C14 | 131.3(1) |
| C1-N1-C2 | 124.4(2) | C13-N3-C14 | 124.3(2) |
| S1-C1-N1 | 125.2(2) | S2-C13-N3 | 126.5(1) |
| O1-C2-N1 | 129.7(2) | O2-C14-N3 | 130.1(2) |

### 3.6. Antitumor evaluation

The acylthiourea ligands and the complexes $\mathbf{Cu(L^1)_2}$, $\mathbf{Cu(L^2)_2}$ and $\mathbf{Cu(L^3)_2}$ were tested *in vitro* for their cytotoxic activity against mouse mammary adenocarcinoma TA3 cell line. Figures 3 and 4 show the concentration-dependent inhibitory effect of ligands and their respective copper (II) complexes on the percentage of cell survival at 48 h of exposure on culture medium. In Figure 3, we can observe that the ligand $\mathbf{HL^3}$ showed a major cytotoxic activity (~ 40 μM) than ligands $\mathbf{HL^1}$ and $\mathbf{HL^2}$ (>160 μM) in the survival range of 50-60 %. This high cytotoxicity that $\mathbf{HL^3}$ presents could be due to the biotransformation of the nitrophenyl group to its nitrophenyl radical anion ($PhNO_2^{\cdot-}$) catalyzed by the enzyme NADPH- cytochrome $P_{450}$ reductase, together with the formation of the superoxide radical anion ($O_2^{\cdot-}$) /47-49/. As we can see in Figure 4, the copper (II) complexes turned out to be highly more cytotoxic than their ligands, at concentration range of 1-10 μM for 48 h of exposure in tumor cells. The complex $\mathbf{Cu(L^3)_2}$ presented a constant decrease of cellular survival by about 15 % higher in comparison with $\mathbf{Cu(L^1)_2}$ and $\mathbf{Cu(L^2)_2}$ in the range of 1-5 μM. The copper complexes show a strong inhibition of the cell proliferation at the concentration of 10 μM, where the cell survival was reduced to 20 % of the control. These results indicate that the chelation of acylthiourea ligands with copper (II) dramatically enhances the cytotoxic activities. Furthermore, the participation of the nitro and chloro substituent groups in the activity of complexes is slightly relevant.

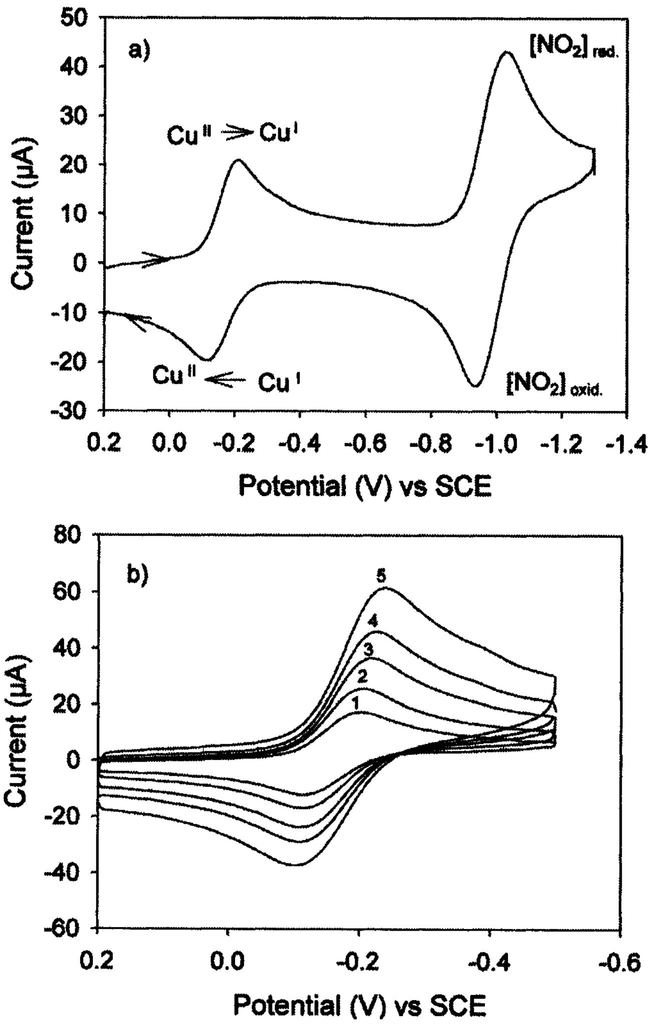

**Fig. 2:** (a) Cyclic voltammogram of the complex $Cu(L^3)_2$ in DMSO. (b) Variation of the copper(II)/ copper(I)-redox peaks with the sweep rate. 1: 50 $mVs^{-1}$; 2: 100 $mVs^{-1}$; 3: 200 $mVs^{-1}$; 4: 300 $mVs^{-1}$; 5: 500 $mVs^{-1}$.

$IC_{50}$ values for the tested compounds are given in Table 3. The ligands showed high $IC_{50}$ values at the range of 40.0 – 240 μM, whereas the complexes $Cu(L^1)_2$, $Cu(L^2)_2$ and $Cu(L^3)_2$ displayed the lowest $IC_{50}$ values (6.93, 5.42 and 3.87 μM, respectively) against to the TA3 tumor cell line. These results were comparable with those of the square-planar copper (II) complexes of thiosemicarbazone and carboxamidazone derivatives ($IC_{50}$ = 12.5 and 3.0 μM, respectively) tested against the MCF-7 human breast adenocarcinoma cell line /50, 51/. On the other hand, the $IC_{50}$ values of these copper (II) complexes resulted to be near to those of the related platinum (II) complexes ($IC_{50}$ = 2.6 – 2.8 μM) /23/ and cisplatin ($IC_{50}$ = 1.3 μM) evaluated in the same tumor cell line. These results demonstrate that the bis-chelate copper complexes, with a slightly distorted square-planar geometry play an important role in the inhibition of TA3 tumor cell growth.

**Table 3**

In vitro cytotoxic activities of the ligands and their copper(II) complexes against the mouse mammary adenocarcinoma TA3 cell line.

| Compound | $IC_{50}$ (μM) [a] |
|---|---|
| $HL^1$ | 240 ± 1.9 |
| $Cu(L^1)_2$ | 6.93 ± 0.05 |
| $HL^2$ | 176.27 ± 1.42 |
| $Cu(L^2)_2$ | 5.42 ± 0.12 |
| $HL^3$ | 40.0 ± 1.1 |
| $Cu(L^3)_2$ | 3.87 ± 0.08 |

[a] $IC_{50}$ corresponds to the concentration required to inhibit 50% of the culture growth when cells are exposed to compounds for 48 h. Each value is the mean ± SD of three independent experiments with each assay performed in triplicate.

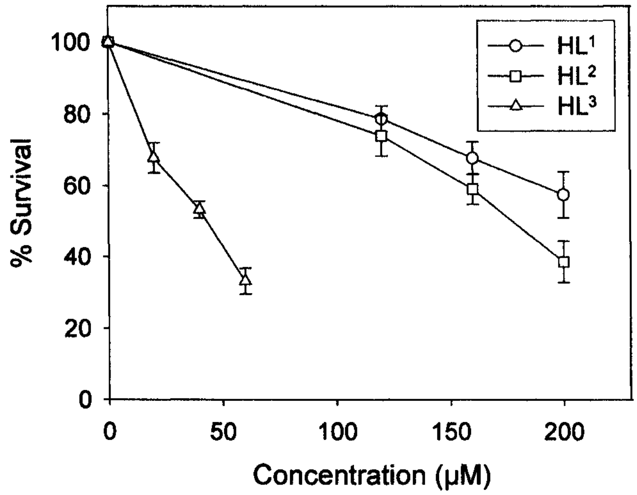

**Fig. 3:** Cytotoxic effect of the ligands $HL^1$, $HL^2$ and $HL^3$ on TA3 tumor cell line cultured during 48 hr. Each value is the mean ± SD of three independent experiments, where each assay was performed in triplicate.

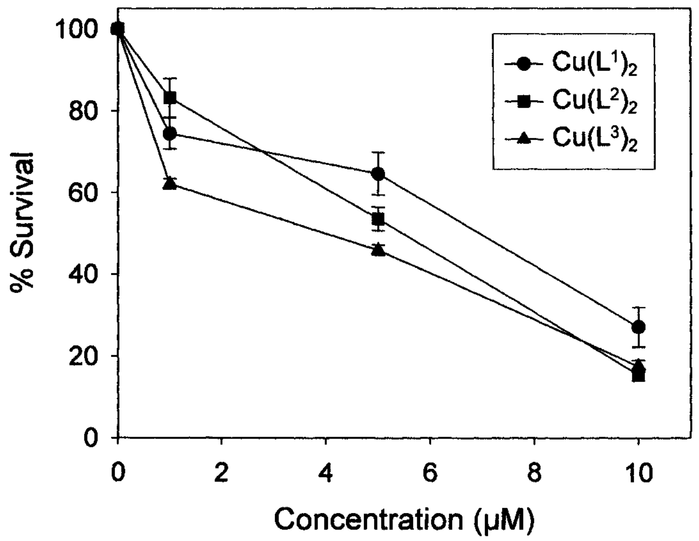

**Fig. 4:** Cytotoxic effect of the complexes $Cu(L^1)_2$, $Cu(L^2)_2$ and $Cu(L^3)_2$ on TA3 tumor cell line cultured during 48 hr. Each value is the mean ± SD of three independent experiments, where each assay was performed in triplicate.

### 3.7. Accumulation of copper in TA3 cells and binding to cellular DNA

As we can see from Figure 5, the complexes $\mathbf{Cu(L^2)_2}$ and $\mathbf{Cu(L^3)_2}$ enter into the cells more rapidly than $\mathbf{Cu(L^1)_2}$. After 2.5 h of exposure, copper accumulated in TA3 cells from complexes $\mathbf{Cu(L^1)_2}$, $\mathbf{Cu(L^2)_2}$ and $\mathbf{Cu(L^3)_2}$ were 7.12, 12.1 and 14.5 nmol Cu/$10^6$ cells, respectively. Significantly, 2.3 times higher $\mathbf{Cu(L^3)_2}$ than $\mathbf{Cu(L^1)_2}$ was accumulated in TA3 cells, whereas $\mathbf{Cu(L^3)_2}$ cellular accumulation was only slightly higher than $\mathbf{Cu(L^2)_2}$. Complexes $\mathbf{Cu(L^2)_2}$ and $\mathbf{Cu(L^3)_2}$ are incorporated in TA3 cells at similar concentrations than platinum(II) complex, $PtNH_3Cl_2(L)$ [L=2-Phenylpyridine] (100 μM, 3 h of exposure) in mouse sarcoma 180 cells (13.5 nmol Pt/$10^6$ cells) /52/. These results indicate that the hydrophobicity of these copper complexes together with the presence of the nitro and chloro substituent groups in the benzoyl moiety seems to expedite the transport of these complexes through the cellular membrane /52, 53/.

Figure 6 shows the number of drug molecules bound per base pair ($r_b$) based on different concentrations of the copper complexes for 2 h of exposure. The complexes $\mathbf{Cu(L^2)_2}$ and $\mathbf{Cu(L^3)_2}$ displayed a higher DNA-binding activity than $\mathbf{Cu(L^1)_2}$. At the concentration range of 20-100 μM, the complex $\mathbf{Cu(L^3)_2}$ binds 2 times more quickly to DNA than $\mathbf{Cu(L^1)_2}$. These results are in accordance with the cytotoxic activities *in vitro* found for these complexes and the differences of binding to DNA that are presented by these copper(II) complexes may be caused by their lipophylic properties, incorporation rate and their square-planar geometry /54/.

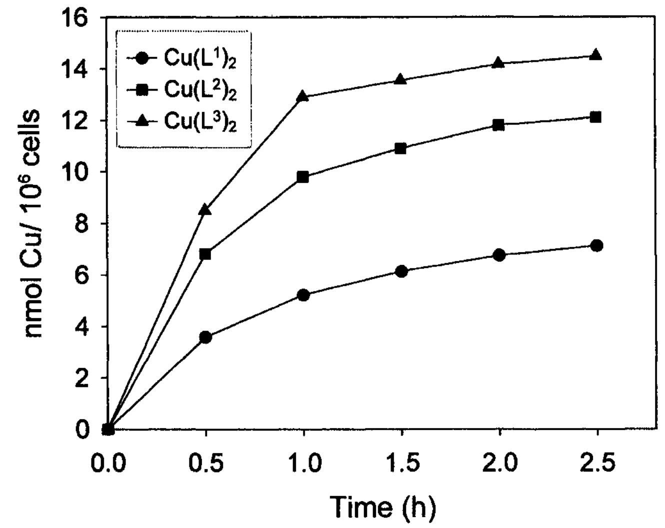

**Fig. 5:** Cellular accumulation of copper as a function of exposure time of the copper(II) complexes to TA3 cells. Data represent the mean value of two independent experiments.

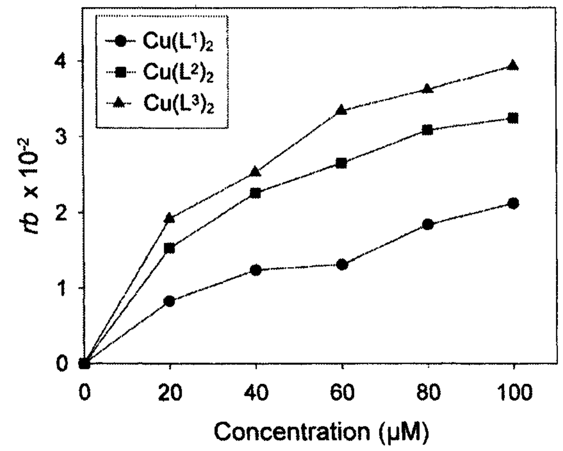

**Fig. 6:** Molecules bound per base pair (*rb*) to DNA isolated from TA3 cells after incubation for 2 h of the copper(II) complexes at different micromolar concentrations. Data represent the mean value of two independent experiments.

### 4. CONCLUSIONS

In summary, we have prepared the bis-chelate copper (II) complexes from acylthiourea derivatives. The crystal structure of $Cu(L^2)_2$ shows that the copper atom presents a nearly square-planar geometry with two bidentate ligands having sulfur and oxygen as donor atoms in *cis* positions.

*In vitro* cytotoxic activity tests against the mouse mammary adenocarcinoma TA3 cell line showed that the copper (II) complexes were more cytotoxic at low micromolar concentrations with respect to the free ligands. In particular the complex $Cu(L^3)_2$ was able to induce a notable decrease of cell survival with a $IC_{50}$

value very similar to those of the related platinum (II) complexes tested under the same experimental conditions. In addition, the complexes **$Cu(L^2)_2$** and **$Cu(L^3)_2$** have shown remarkable copper accumulation in TA3 cells and higher binding to cellular DNA than **$Cu(L^1)_2$**. We hope the present results may be complemented with other additional biological tests involving the assessment of their cytotoxic effects on different tumor cell lines, their toxicity in vivo and the biodistribution of these compounds in diverse organs with the purpose to lead to the development of a new class of antitumor agents.

### 5. SUPPLEMENTARY MATERIAL

Further details of the crystal structure determination are available on request from the Cambridge Crystallographic Data Center (CCDC, 12 Union Road, Cambridge CB2 1EZ, UK; fax: +44 1223 336033; e-mail: deposit@ccdc.cam.ac.uk), on quoting the depositing number CCDC-234020, the names of the authors, and the journal citation.

### ACKNOWLEDGEMENTS

W. H. thanks DAAD for the A/01/176226 doctoral scholarship granted in Chile. This study was also supported by Departamento de Post-grado y Postítulo, Universidad de Chile (PG/91/2002) and FONDAP 11980002 project.

### REFERENCES

1. B. Rosenberg. In: *Metal Ions in Biological Systems*. Sigel H. Ed., New York, vol II, p.127 (1980).
2. T. Hambley, *Coord. Chem. Rev.*, **166**, 181 (1997).
3. M. Nicolini and L. Sindellari. *Lectures in Bioinorganic Chemistry*. Cortina Raven Ed., Verona, Italy, pp.25-30 (1991).
4. R. Brockman, J. Thompson, M. Bell and H. Skipper, *Cancer Res.*, **16**, 167 (1956).
5. W. Antholine, B. Kalyanaraman and D. Petering, *Environ. Health Perspect.*, **64**, 19 (1985).
6. H. Tamura and H. Imai, *J. Am. Chem. Soc.*, **109**, 6870 (1987).
7. J. Dimmock, S. Pandeya, J. Quil, U. Pugazhenthi, T. Allen, G. Kao, J. Balzarine and E. Declercq, *Eur. J. Med. Chem.*, **30**, 655 (1995).
8. R. Casero, D. Klayman, G. Childs, J. Scovill and R. Desjardins, *Antimicrob. Agents Chemother.*, **18**, 317 (1980).
9. J. Patole, U. Sandbhor, S. Padhye, D. Deobagkar, C. Anson and A. Powell, *Bioorg. Med. Chem. Lett.*, **13**, 51 (2003).
10. J. Easmon, G. Purstinger, G. Heinisch, T. Roth, H. Fiebig, W. Holzer, W. Jager, M. Jenney and J.

Hofmann, *J. Med. Chem.*, **44**, 2164 (2001).

11. I. Hall, O. Wong and J. Chapman, *Anticancer Drugs*, **6**, 147 (1995).
12. W. Antholine, J. Knight and D. Petering, *J. Med. Chem.*, **19**, 399 (1976).
13. S. Jayasree and K. Aravindakshan, *Polyhedron*, **12**, 1187 (1993).
14. W. Antholine, J. Knight, H. Whelan and D. Petering, *Mol. Pharmacol.*, **13**, 89 (1977).
15. L. Saryan, E. Ankel, C. Krishnamurti, D. Petering and H. Elford, *J. Med. Chem.*, **22**, 1218 (1979).
16. W. Antholine, P. Gunn and L. Hopwood, *Int. J. Radiat. Oncology, Biol. Phys.*, 7, 491 (1981).
17. S. Jayasree and K. Aravindakshan, *Transition Met. Chem.*, **18**, 85 (1993).
18. E. Blanz and F. French, *Cancer Res.*, **28**, 2419 (1965).
19. F. French, E. Blanz, J. Do Amaral and D. French, *J. Med. Chem.*, **13**, 1117 (1970).
20. B. B. Mohapatra, S. Guru and B.K. Mohapatra, *J. Inorg. Nucl. Chem.*, **39**, 2291 (1977).
21. W. Antholine and F. Taketa, *J. Inorg. Biochem.*, **16**, 145 (1982).
22. X. Shen, J. Shan, H. Sun and B. Kang, *J. Chinese. Chem. Soc.*, **46**, 179 (1999).
23. W. Hernández, E. Spodine, J.C. Muñoz, L. Beyer, U. Schröder, J. Ferreira and M. Pavani. *Bioinorg. Chem. Appl.*, **1**, 271 (2003).
24. A. Earnshaw. *Introduction to Magnetochemistry*. Academic Press, London and N. York, pp.4-8 (1968).
25. H. Hartmann and I. Reuther, *J. Prakt. Chem.*, **315**, 144 (1973).
26. J. Brindley, J. Caldwell, G. Meakins, S. Plackett and S. Price, *J. Chem. Soc. Perkin I*, 1153 (1987).
27. G. M. Sheldrick, SHELXS 97, Program for Solving Crystal Structures, University of Göttingen, Germany 1997.
28. G. M. Sheldrick, SHELXL 97, Program for Structure Refinement, University of Göttingen, Germany 1997.
29. J. Ferreira, L. Coloma, E. Fones, M. Letelier, Y. Repetto, A. Morello and J. Aldunate, *FEBS Lett.*, **234**, 485 (1988).
30. K. Skov, H. Adomat, N. Farrell and J. Matthews, *Anti-cancer Drug Des.*, **13**, 207(1998).
31. B. Jansen, J. van der Zwan, H. den Dulk, J. Brouwer and J. Reedijk, *J. Med. Chem.* **44**, 45(2001).
32. A. Mohamadou, I. Déchamps-Oliver and J. Barbier, *Polyhedron*, **13**, 3277 (1994).
33. C. Sacht, M. Datt, S. Otto and A. Roodt, *J. Chem. Soc. Dalton Trans.*, 727 (2000).
34. E. Guillon, A. Mohamadou, I. Déchamps-Oliver and J. Barbier, *Polyhedron*, **15**, 947 (1996).
35. L. Beyer, E. Hoyer, L. Liebscher and H. Hartmann, *Z. Chem.*, **21**, 81 (1980).
36. K.R. Koch, A. Irving and M. Matoetoe, *Inorg. Chim. Acta*, **206**, 193 (1993).
37. K.R. Koch, C. Sacht and S. Bourne, *Inorg. Chim. Acta*, **232**, 109 (1995).
38. C. Sacht and M.S. Datt, *Polyhedron*, **19**, 1347 (2000).
39. C. Sacht, M.S. Datt, S. Otto and A. Roodt, *J. Chem. Soc. Dalton Trans.*, 4579 (2000).
40. A. Mohamadou, I. Déchamps-Oliver and J. Barbier, *Polyhedron*, **13**, 1363 (1994).
41. F. Leβmann, Doctoral Tesis, University of Leipzig, Germany, **1999.**
42. E. Guillon, I. Déchamps-Olivier, A. Mohamadou and J.-P. Barbier, *Inorg. Chim. Acta*, **268**, 13 (1998).
43. L. Beyer, E. Hoyer, H. Hennig, R. Kirmse, H. Hartmann and J. Liebscher, *J. prakt. Chem.*, **317**, 829 (1975).

44. R. Richter, L. Beyer and J. Kaiser, *Z. anorg.allg. Chem.*, **461**, 67 (1980).
45. J. Gomes and M. Ribeiro da Silva, *Inorg. Chem. Comm.*, **6**, 149 (2003).
46. R. Bailey, K. Rothaupt and R. Kullnig, *Inorganica Chimica Acta*, **147**, 233 (1988).
47. M. Virginia and R. Mason, *J. Biol. Chem.*, **264**, 12379(1989).
48. H. Halliwell and C. Cross, *J. Lab. Clin. Med.*, **119**, 598(1992).
49. M. Olmstead, R. Hamilton and R. Nicholson, *Analytical Chemistry*, **41**, 260(1969).
50. A. Murugkar, S. Padhye, S. Guha-Roy and U. Wagh, *Inorg. Chem. Comm.*, **3**, 545 (1999).
51. N. Gokhale, S. Padhye, D. Rathbone, D. Billington, P. Lowe, C. Schwalbe and C. Newton, *Inorg. Chem. Comm.*, **4**, 453 (1999).
52. T. Okada, I. El-Mehasseb, M. Kodaka, T. Tomohiro, K. Okamoto and H. Okuno, *J. Med. Chem.*, **44**, 4661(2001).
53. E. Khazanov, Y. Barenholz, D. Gibson and Y. Najajreh, *J. Med. Chem.*, **45**, 5196(2002).
54. T. Tanaka, K. Tsurutani, A. Komatsu, T. Ito, K. Iida, Y. Fujii, Y. Nakano, Y. Usui, Y. Fukuda and M. Chikira, *Bull. Chem Soc. Jpn.*, **70**, 615(1997).

